# Systemic Inflammation Index Values Are Associated With Worsened Disease Severity and Poor Response to Autoimmune Encephalitis Treatment

**DOI:** 10.3389/fneur.2021.709553

**Published:** 2021-10-05

**Authors:** Yanliang Mei, Jing Yang, Yanpeng Yuan, Yutao Liu, Xiaojing Liu, Mingli Li, Shiheng Fan, Lanjun Li, Chenyang Jiang, Yuming Xu

**Affiliations:** ^1^Department of Neurology, The First Affiliated Hospital of Zhengzhou University, Zhengzhou, China; ^2^National Health Commission Key Laboratory of Cerebrovascular Disease, Zhengzhou University, Zhengzhou, China

**Keywords:** autoimmune encephalitis, immunotherapy, response to treatment, systemic inflammation index, immune responses

## Abstract

Both specific and innate immune responses play important roles in autoimmune encephalitis (AE). We aimed to explore the predictive value of the systemic inflammation index (SII) at admission as a peripheral biomarker of treatment response of AE. A total of 146 patients diagnosed with AE in the First Affiliated Hospital of Zhengzhou University from January 1, 2018 to September 22, 2020 were retrospectively and consecutively analyzed as per the inclusion criteria and divided into two groups according to their response to immunotherapy after 30 days. The predictive value of the SII as a peripheral biomarker for AE treatment response was calculated using the receiver operating characteristic curve analysis, which showed that the best SII cut-off value for predicting poor response to AE treatment was 863.3; the area under the curve was 0.75, with 83.0% sensitivity and 72.0% specificity. The risk factors for poor response to AE treatment were analyzed; univariable analysis showed that the rate of decreased level of consciousness, rate of cognitive or mental behavior abnormality, cerebrospinal fluid pressure, blood neutrophils, platelets, time until treatment initiation, neutrophil to lymphocyte ratio, platelet to lymphocyte ratio, and SII were significantly higher in patients with poor response to AE immunotherapy after 30 days than in patients with good response. Meanwhile, the blood lymphocyte counts and Glasgow Coma Scale (GCS) scores in patients with poor response were significantly lower than those in patients with good response (all *p* < 0.05), and multivariable binary logistic regression with backward stepwise method showed that decreased levels of consciousness, time until treatment initiation and SII were associated with poor response to immunotherapy. Moreover, the SII ≤ 863.3 group had lower rates of decreased consciousness levels, admission to the intensive care unit, and mechanical ventilation; lower cerebrospinal fluid pressure, blood neutrophil count, and platelet count; and higher blood lymphocyte count and GCS scores. The SII was associated with worsened disease severity and poor response to treatment after 30 days of the initially diagnosed AE, and patients with an SII > 863.3 were more likely to have poor response to immunotherapy.

## Introduction

Autoimmune encephalitis (AE) refers to encephalitis caused by an immune response to the central nervous system antigens mediated by autoimmune mechanisms. AE can occur at all ages and carries a serious burden for patients and society. Currently, AE mainly refers to encephalitis related to antibodies to neuronal cell-surface or synaptic receptors. The main clinical symptoms include abnormal behavior or cognitive dysfunction, speech dysfunction, seizures, dyskinesias, movement disorders, decreased levels of consciousness, and autonomic dysfunction (1, 2). Since the discovery of anti-N-methyl-D-aspartate receptor (anti-NMDAR) antibodies (3), other AE-related antibodies have been discovered (4), such as anti-leucine-rich glioma-inactivated 1 (anti-LGI1) antibody, anti-γ-aminobutyric acid B receptor (anti-GABA_B_R) antibody, AMPA-type glutamate receptors (AMPAR), dipeptidyl-peptidase-like protein-6 (DPPX), glutamic acid decarboxylase (GAD), and anti-contactin-associated protein-like 2 (anti-CASPR2) antibody, among which the anti-NMDAR antibody is the most common (5, 6).

AE has complex and severely disabling clinical manifestations, and patients may differ in their response to immunotherapy (7). No biomarkers that can effectively predict the response to immunotherapy of patients with AE have been found. Therefore, detecting such markers would be especially important for the clinical treatment and management of AE.

The specific immune response mediated by antibodies to neuronal cell-surface or synaptic receptors is one of the main pathogenic mechanisms and is a pathological feature of AE; however, innate immunity also plays a role in the pathogenesis of this disorder (8). Previous studies have shown that monocyte infiltration and microglia proliferation, both pathological features of AE, lead to blood-brain barrier dysfunction, which in turn activates the adaptive immune response (9). In addition, studies have shown that innate immune cells such as monocytes (10) play an important role in neuroinflammation. Starossom et al. (11) showed that platelets have a two-way regulatory role in central nervous system inflammation.

The inflammatory response in the body is mainly manifested as changes in related inflammatory proteins and inflammatory cell counts in peripheral blood. The most common markers used to assess the state of inflammation of the body include the neutrophil-lymphocyte ratio (NLR), C-reactive protein-albumin ratio (CAR), platelet-lymphocyte ratio (PLR), lymphocyte to monocyte ratio (LMR), systemic inflammatory index (SII), and others. Some previous studies have shown that the SII is related to the severity and prognosis of acute stroke (12). Moreover, some studies have pointed out that blood inflammatory markers are related to some autoimmune diseases (13, 14). Both specific and innate immune responses play important roles in AE (8); however, few reports exist on the predictive value of blood inflammatory markers for treatment response. Therefore, this study aimed to evaluate the correlation between the SII and other related inflammatory markers with treatment response at 30 days in patients with acute AE. We also aimed to determine if the SII could be used as an independent predictor of response to treatment.

## Subjects and Methods

### Patient Characteristics

We retrospectively and consecutively included patients who were diagnosed with AE at the First Affiliated Hospital of Zhengzhou University from January 1, 2018, to September 22, 2020. The study protocol was approved by the human ethics committee at the First Affiliated Hospital of Zhengzhou University and followed the Declaration of Helsinki. Good response to treatment was defined as a modified Rankin scale (mRS) score at 30 days of first-line immunotherapy lower than that at presentation; an unchanged or higher mRS score was defined as poor response. The standard treatment for the two groups was first-line immunotherapy including methylprednisolone (1 g daily for 3 days; 500 mg daily for 3 days; and then oral prednisone) in addition to intravenous immunoglobulin (2 g/kg over 5 days at 400 mg/kg/day) or plasma exchange (1 session on alternate days for 5 cycles) (15). None of AE patients received second-line therapy (rituximab, cyclophosphamide, or other) within 30 days of first-line immunotherapy. The inclusion criteria were as follows: (1) over 18 years old, (2) meeting the diagnostic criteria of AE established by Mittal and Graus et al. (16), (3) testing positive for AE-related antibodies in blood or cerebrospinal fluid, and (4) initially diagnosed with AE and having received first-line immunotherapy (15). The exclusion criteria were as follows: (1) other acute neurological diseases, such as viral encephalitis, etc., (2) previous physical disability symptoms, (3) comorbidity with other autoimmune diseases, (4) comorbidity with neoplastic or hematological diseases, (5) infectious diseases such as respiratory or genitourinary system infection at the time of admission, or (6) having received, before presentation, immunosuppressive drugs potentially affecting the number of blood immune cells.

### Hematological Analysis

The results of the most recent blood routine tests, blood biochemistry, cerebrospinal fluid, cerebral imaging, and other related examinations following admission to the hospital were retrieved from the electronic medical record system, together with other patient baseline data, such as clinical symptoms, immunotherapy methods, and mRS at admission. The mRS scores at 30 days after immunotherapy were also collected. The tests used to assess the NLR, PLR, LMR, and SII were all performed within 24 h of admission and before immunotherapy. The NLR was calculated as neutrophil count (/L)/lymphocyte count (/L); the PLR was calculated as platelet count (/L)/lymphocyte count (/L); the LMR was calculated by lymphocyte count (/L)/monocyte count (/L); the SII was calculated as platelet count (/L)×neutrophil count (/L)/lymphocyte count (/L).

### Statistical Analysis

All data were analyzed using Stata 16.0 software (StataCorp LLC, TX, USA). Continuous variables are reported as mean ± standard deviation (SD) or median and analyzed by the independent Student's *t*-test or the Mann-Whitney test as appropriate. Categorical variables are reported as numbers and analyzed using the chi-square test or the Fisher exact test. Binary logistic regression analysis was used to identify independent risk factors for treatment response in patients with AE. And the candidate variables with a univariate relationship (*P* < 0.25) with outcome or considered clinical relevant were selected as inputs into a multivariate logistic regression model with backward stepwise method. The performance of logistic model was assessed by the area under the ROC curve (AUC) and Hosmer-Lemeshow goodness-of-fit test. *P*-values < 0.05 were considered statistically significant. Receiver operating characteristic (ROC) curve analysis was used to explore the value of the SII as a predictor of treatment response in AE.

## Results

### Baseline Characteristics and Univariable Analysis of Factors Associated With Poor Treatment Response

We included 165 patients with confirmed AE in our initial cohort. Two patients were excluded because they had severe respiratory infection. Six patients were excluded due to the absence of laboratory data. Five patients with malignant tumors and six who refused immunotherapy were also excluded. Finally, a total of 146 patients with AE (80 NMDAR, 36 LGI-1 and 30 other antibody types) were enrolled, with an average age 41.7 ± 18.4 years, including 79 men (54.1%) and 67 women (45.9%). At 30 days after immunotherapy, 88 (60.3%) patients had good treatment response and 58 (39.7%) had poor treatment response. Univariable analysis showed that compared with the good response group, the poor response group had higher rates of decreased level of consciousness, abnormal behavior or cognitive dysfunction; higher cerebrospinal fluid pressure, blood neutrophil count, time until treatment initiation, platelets, alanine transaminase (ALT), NLR, PLR, and SII; and lower blood lymphocyte count and GCS scores (Table 1).

**Table 1:** Comparison of baseline characteristics of patients with different treatment responses.

**Variables**	**Good response**	**Poor response**	***P-*** **value**
Age (years)	40.93 ± 18.09	42.88 ± 19.00	0.536
Male sex	56.8%	50.0%	0.419
Fever (>37.5°C)	37.5%	29.3%	0.308
Speech dysfunction	15.9%	22.4%	0.322
Decreased level of consciousness	25.0%	67.2%	0.001
Treatment response	88	58	/
**mRS scores**			
0–2	17	13	
3–6	71	45	0.651
GCS scores	12.44 ± 2.3	11.12 ± 2.7	0.002
Autonomic dysfunction	17.2%	19.3%	0.754
Seizures	51.1%	62.1%	0.193
Abnormal behavior or Cognitive dysfunction	72.7%	87.9%	0.028
Movement disorders	27.2%	39.8%	0.117
Time until treatment initiation	28.24 ± 19.98	36.08 ± 23.14	0.015
Abnormal MRI	36.4%	43.1%	0.842
CSF pressure (mmH2O)	166.7 ± 50.3	192.6 ± 77.6	0.020
CSF WBC count (/L)	16.1 ± 28.0	14.7 ± 18.9	0.370
CSF protein (mg/L)	394.9 ± 218.7	401.6 ± 327.3	0.442
ESR (mm/h)	9.7 ± 10.0	12.7 ± 13.1	0.080
Creatinine	64.3 ± 26.3	58.6 ± 14.2	0.068
Uric acid	263.3 ± 211.1	237.1 ± 124.4	0.799
ALT (U/L)	23.8 ± 21.3	37.9 ± 46.8	0.008
AST (U/L)	23.1 ± 23.7	38.7 ± 66.1	0.060
Serum albumin	42.7 ± 4.6	42.3 ± 5.3	0.303
Hemoglobin	146.6 ± 130.1	131.5 ± 27.5	0.193
Platelets	230.5 ± 58.7	254.6 ± 82.3	0.021
WBC (/L)	8.4 ± 3.7	10.3 ± 8.5	0.033
Neutrophil count (/L)	5.9 ± 3.5	8.2 ± 7.6	0.008
Lymphocyte count (/L)	1.8 ± 0.7	1.5 ± 0.6	0.003
Monocyte count (/L)	0.6 ± 0.3	0.6 ± 0.3	0.510
Eosinophil count (/L)	0.08 ± 0.07	0.09 ± 0.1	0.474
Basophil count (/L)	0.03 ± 0.03	0.04 ± 0.05	0.598
NLR	4.1 ± 5.3	6.9 ± 6.0	0.003
PLR	150.3 ± 76.4	209.7 ± 128.8	0.001
SII	937.7 ± 1229.6	1615.9 ± 1301.4	0.001

*Data are shown as mean ± standard deviation or as percentage. MRI, magnetic resonance imaging; CSF, cerebral spinal fluid; WBC, white blood cell; ESR, erythrocyte sedimentation rate; ALT, alanine transaminase; AST, aspartate transaminase; NLR, neutrophil-lymphocyte ratio; PLR, platelet-lymphocyte ratio; SII, systemic inflammation index; mRS; modified Rankin scale; GCS; Glasgow Coma Scale*.

### The Predictive Value of the SII on the Response to Treatment

**Figure 1:**
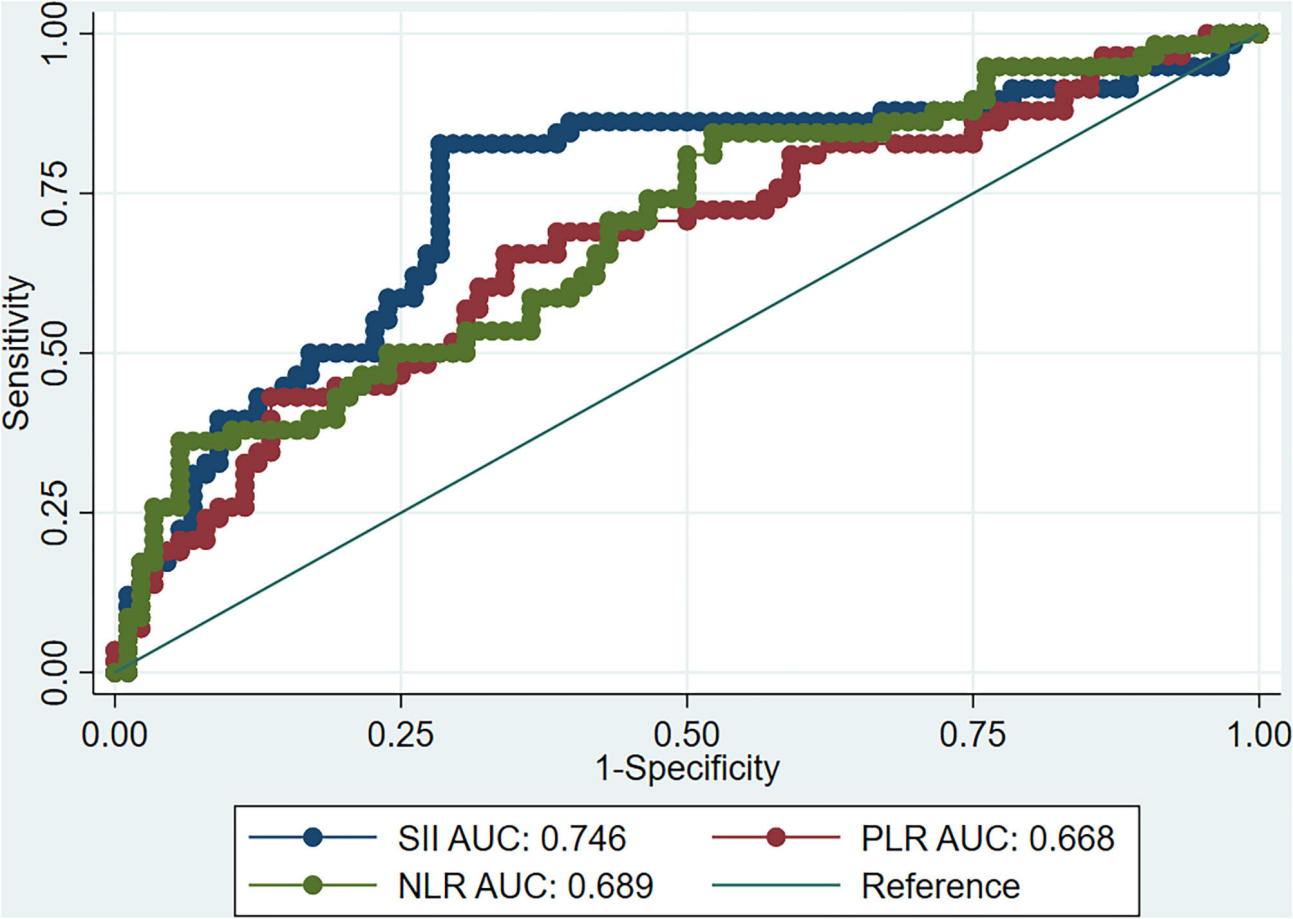
ROC curve of SII, NLR, and PLR as predictors of the response of AE to immunotherapy. ROC, receiver operating characteristic; SII, systemic inflammation index; NLR, neutrophil-lymphocyte ratio; PLR, platelet-lymphocyte ratio; AE, autoimmune encephalitis.

ROC curve analysis showed that the best SII cut-off value for predicting poor response to AE treatment after 30 days was 863.3, and the area under the curve (AUC) was 0.75 [95% confidence interval (CI) 0.66–0.83], with 83.0% sensitivity and 72.0% specificity. We also calculated the predictive value of NLR and PLR on the treatment response. The AUC of NLR was 0.69, with 81.0% sensitivity, 50.0% specificity, and a best cut-off value of 2.89. The AUC of PLR was 0.67, with 66.0% sensitivity, 66.0% specificity, and a best cut-off value of 150.68 (Figure 1). The ROC curve analysis showed a relatively lower specificity of the SII and NLR values, indicating the presence of a false-positive population of AE patients who had higher scores yet a favorable response to therapy.

### Multivariable Logistic Analysis of the Predictors of Poor Treatment Response

After correcting for confounding factors, the multivariable logistic analysis found that an SII ≥ 863.3 [odds ratio (OR) 28.04, 95% CI 5.23–150.17, *P* = 0.001], time until treatment initiation (OR 1.03, 95% CI 1.01–1.05, *P* = 0.008) and decreased level of consciousness (OR 10.15, 95% CI 3.71–27.76, *P* = 0.001) were associated with poor response to first-line immunotherapy at 30 days. However, high PLR, high NLR, lower peripheral blood lymphocyte count, higher peripheral blood neutrophil count, and abnormal behavior or cognitive dysfunction were not associated with poor response (Table 2). This logistic model had an area under the curve of 0.89 and no significant lack of fit (Hosmer-Lemeshow goodness-of-fit test *P* = 0.47).

**Table 2:** Multivariable analysis of treatment response.

**Variable**	**Values**	**Odds ratio**	**95% CI**	***P*** **-value**
WBC	/L	1.05	0.98–1.12	0.161
Time until treatment initiation	Days	1.03	1.01–1.05	0.008
Decreased level of consciousness	Yes vs. no	10.15	3.71–27.76	0.001
Abnormal behavior or cognitive dysfunction	Yes vs. no	2.33	0.62–8.75	0.211
NLR	≥2.89 vs. <2.89	0.20	0.04–1.15	0.071
SII	≥863.31 vs. <863.31	28.04	5.23–150.17	0.001

*WBC, White blood cell; NLR, neutrophil to lymphocyte ratio; SII, systemic inflammation index*.

### Subgroup Analysis of the Predictive Value of the SII in Different Types of AE

**Figure 2:**
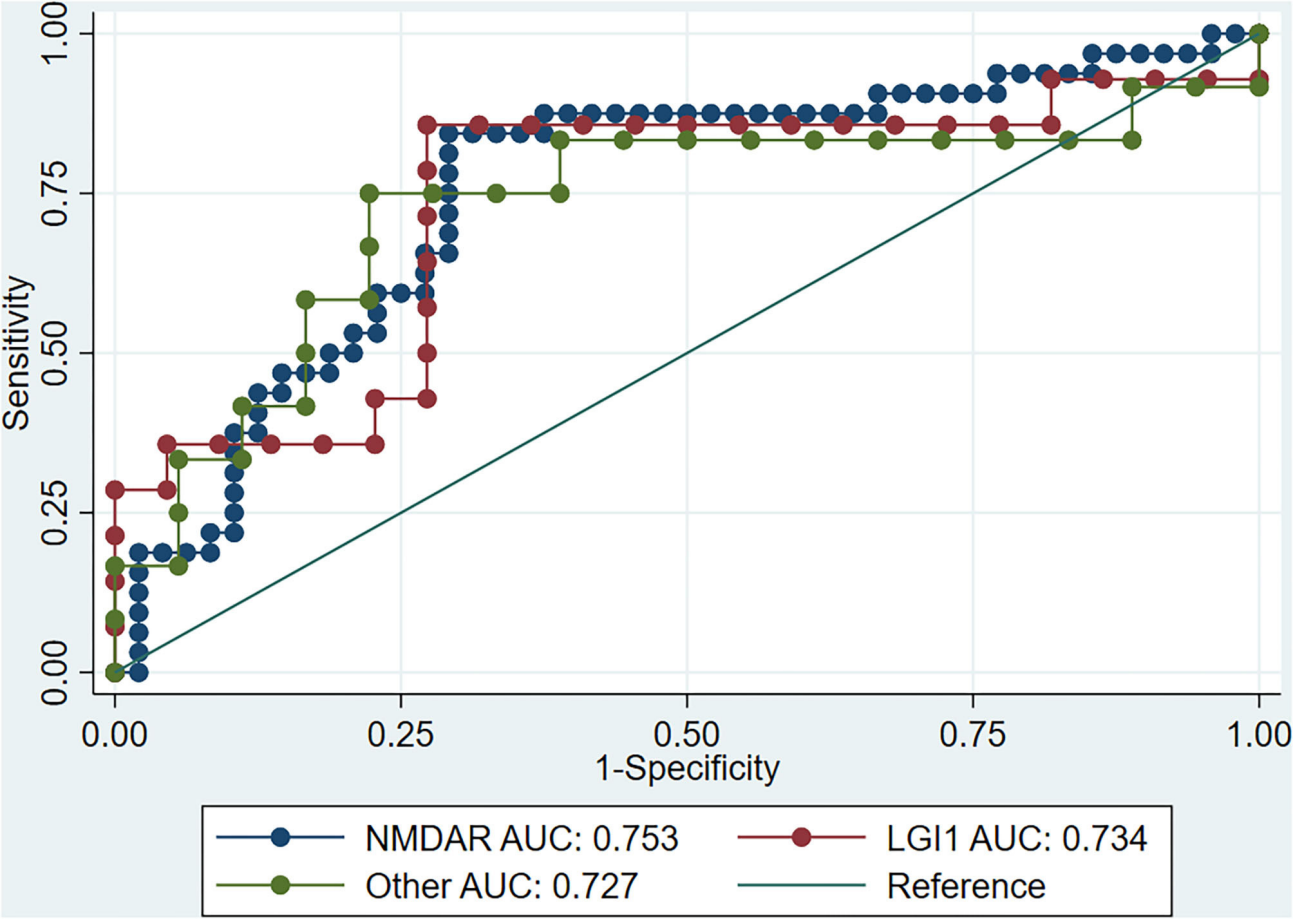
ROC curve of SII for immunotherapy response in different AE types. ROC, receiver operating characteristic; SII, systemic inflammation index; AE, autoimmune encephalitis; NMDAR, N-methyl-D-aspartate receptor; LGI1, leucine-rich glioma-inactivated 1.

In order to explore the predictive value of the SII for treatment response in different types of AE, we carried out a subgroup analysis based on antibody types. A total of 146 patients with AE were enrolled, with 80 NMDAR antibody type, 36 LGI-1 antibody type and 30 other AE antibody type (AMPAR = 2, DPPX = 1, CASPR2 = 10, GABA_B_R = 14, GAD = 2, GFAP = 1). Such analysis showed that the SII had good predictive value for NMDAR antibody encephalitis, anti-LGI1 encephalitis, and other types of AE (Table 3). Moreover, there was no significant difference in the predictive value of the SII for response to treatment of AE with different antibody types (*P* > 0.05, Figure 2).

**Table 3:** Predictive value of SII for treatment responses in different types of AE.

**AE type**	**AUC**	**95% CI**	**Sensitivity**	**Specificity**	***P*** **-value**
NMDAR	0.75	0.64–0.87	84.0%	71.0%	0.001
LGI1	0.73	0.55–0.92	86.0%	73.0%	0.019
Others	0.73	0.51–0.94	75%	78.0%	0.038

*SII, systemic inflammation index; AE, autoimmune encephalitis; AUC, area under the curve; CI, confidence interval; NMDAR, -N-methyl-D-aspartate receptor; LGI1, leucine-rich glioma-inactivated 1*.

### Relationship Between the SII and Clinical Factors Related to Response to Immunotherapy

According to the SII value, the patients were divided into two groups (SII value ≤ 863.3 and > 863.3), and the clinical factors related to treatment response were compared between the two groups. The results showed that the SII ≤ 863.3 group had lower rate of decreased consciousness levels, admission to the intensive care unit (ICU), and mechanical ventilation; lower cerebrospinal fluid pressure, blood neutrophil count, and platelet count; and higher blood lymphocyte count and GCS scores (all *P* < 0.05; Table 4).

**Table 4:** Relationship between SII and clinical indexes in patients with AE.

**Variables**	**SII ≤ 863.3**	**SII > 863.3**	***P*** **-value**
Decreased level of consciousness	28.80%	54.80%	0.001
GCS scores	12.7 ± 2.1	11.2 ± 2.8	0.006
ICU admission	13.70%	50.68%	0.001
Mechanical ventilation	6.85%	30.14%	0.001
CSF pressure	162.8 ± 44.3	191.2 ± 76.0	0.004
Platelet count	218.7 ± 57.6	261.5 ± 74.5	0.001
Neutrophil count	4.8 ± 1.9	8.8 ± 7.2	0.001
Lymphocyte count	1.96 ± 0.66	1.35 ± 0.58	0.001

*SII, systemic inflammation index; AE, autoimmune encephalitis; GCS; Glasgow Coma Scale; ICU, intensive care unit; CSF, cerebrospinal fluid*.

## Discussion

AE is a disabling immune-inflammatory disease of the nervous system (7). Most patients have good prognosis after active immunotherapy; however, disabling symptoms persist in some patients (17). Previous studies have confirmed the important role of inflammation and the immune response in the occurrence and development of AE (8, 18), and the SII, as an inflammatory marker of peripheral blood, can reflect, to a certain extent, the state of the body's inflammatory immune response.

In view of the importance of inflammation and immune response to the occurrence and development of AE, this study focused on whether SII values were associated with worsened disease severity and poor response to treatment at 30 days of the initially diagnosed AE. The results showed that the SII value significantly correlated with treatment response. ROC curve analysis with the Youden index method found an optimal SII cut-off value of 863.3, with an AUC of 0.75 (95% CI 0.64–0.81), 83.0% sensitivity, and 72.0% specificity, demonstrating the predictive value of the SII. The incidence of poor response to treatment in the SII high-risk group was 65.8%, which was significantly higher than that in the low-risk group (13.7%; *P* < 0.001). After multivariable analysis, it was found that an SII value of >863.3 was associated with poor response to first-line immunotherapy (*P* < 0.05).

The SII is calculated by multiplying the platelet count (/L) by the neutrophil count (/L) divided by the lymphocyte count (/L). The exact biological mechanism by which elevated SII leads to poor response to first-line immunotherapy is still unclear but it is speculated to be related to the factors discussed below.

The blood-brain barrier is an important physiological barrier protecting the central nervous system from inflammatory factors present in the peripheral blood (19). The impairment of blood-brain barrier function is one of the important early features of central nervous system inflammatory immune diseases, such as AE, multiple sclerosis, and optic neuromyelitis (20). Neutrophils play an important role in the occurrence and development of central nervous system inflammation. Studies have shown that neutrophils can release a large number of cytokines, such as interleukin 1 beta (IL-1β), IL-6, and tumor necrosis factor alpha (TNF-α), in the acute phase of central nervous system immune inflammation (21). These cytokines can damage the function of the blood-brain barrier and increase its permeability. In the early stage of immune-inflammatory diseases of the central nervous system, neutrophils can also infiltrate the central nervous system, triggering and aggravating its inflammatory response (22).

Many previous studies have shown that platelets also play an important role in inflammation, especially in some autoimmune diseases, such as arthritis and rheumatoid arthritis (23, 24). However, platelets also play an important role in inflammation of the nervous system (25), and can interact with many types of cells in peripheral blood, including white blood cells. In addition, activated platelets can express selectin and CD40L, as well as inflammatory cytokines and chemokines, to promote the activation of neutrophils (26). Activated platelets can also promote the activation of monocytes and dendritic cells through CD40-CD40L interaction, which promotes antigen presenting cells to transmit antigen information to T cells, thereby enhancing the adaptive immune response (26). Starossom et al. found an important regulatory role of platelets in the inflammatory immune response of the central nervous system (11).

Lymphocytes also play an important role in the occurrence and development of AE. Lymphocytes in peripheral blood can migrate into the central nervous system through a damaged blood-brain barrier and promote inflammation. Studies have shown that the inhibition of lymphocyte migration into the nervous system can effectively inhibit neuroinflammation (27).

In summary, an SII value of >863.3 was associated with worsened disease severity and poor response to immunotherapy at 30 days of the initially diagnosed AE, and the predictive value of the SII holds for patients with different antibody types. Moreover, SII is a widely available blood test which can reflect specific immune response and innate immunity in AE patients. So peripheral SII can be used as an easily measurable and potential biomarker to predict the disease progression of AE, which may be associated with monitoring of disease activity and whether intensive immunosuppressive therapy should be initiated. However, our study is a moderate-sized retrospective cohort and to better understand the predictive value of the SII, we need to investigate more inflammatory factors, increase the sample size, and conduct a prospective randomized controlled trial to confirm our conclusion in this study.

## Data Availability Statement

The raw data supporting the conclusions of this article will be made available by the authors, without undue reservation.

## Author Contributions

JY and YX planned and conceived the study. YM and YY collected the data. XL, ML, and SF interpreted the data. YM and JY wrote and critically revised the manuscript. All authors have read and approved the final manuscript.

## Funding

This work was supported by grants from the National Natural Science Foundation of China to YX (81530037), the National Natural Science Foundation of China to JY (81600946), the Provincial and Ministry of Health Construction Committee of Henan Province to JY (SB201902012), and the Non-profit Central Research Institute Fund of Chinese Academy of Medical Sciences (2020-PT310-01).

## Conflict of Interest

The authors declare that the research was conducted in the absence of any commercial or financial relationships that could be construed as a potential conflict of interest.

## Publisher's Note

All claims expressed in this article are solely those of the authors and do not necessarily represent those of their affiliated organizations, or those of the publisher, the editors and the reviewers. Any product that may be evaluated in this article, or claim that may be made by its manufacturer, is not guaranteed or endorsed by the publisher.
